# Supplementary material for: Identifying repeat domains in large genomes
Source: Genome Biol. 2006 Jan 31;7(1):R7. doi: 10.1186/gb-2006-7-1-r7 (PMC1431705; doi:10.1186/gb-2006-7-1-r7)
Supplement: Additional File 1 — A zipped file of browsable HTML files with a complete list of the connected components in the repeat domain graph of human Repbase. [file gb-2006-7-1-r7-S1.gz › html/subgraphs/8845.pdf]

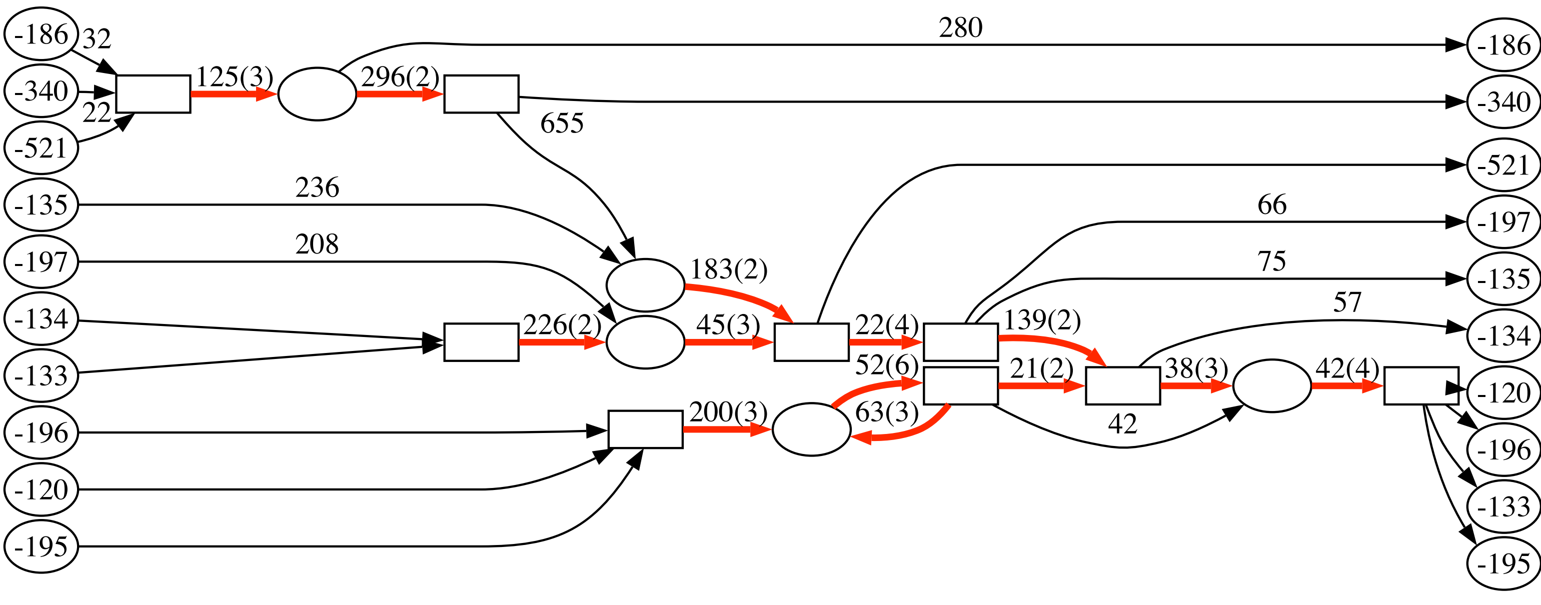
-186
32
-340
22
-521
125(3)
296(2)
280
655
-135
236
-197
208
-134
-133
226(2)
183(2)
45(3)
22(4)
139(2)
21(2)
52(6)
63(3)
38(3)
42(4)
42
66
75
57
-196
-120
-195
200(3)
-186
-340
-521
-197
-135
-134
-120
-196
-133
-195
